# SpectroPipeR—a streamlining post Spectronaut^®^ DIA-MS data analysis R package

**DOI:** 10.1093/bioinformatics/btaf086

**Published:** 2025-02-22

**Authors:** Stephan Michalik, Elke Hammer, Leif Steil, Manuela Gesell Salazar, Christian Hentschker, Kristin Surmann, Larissa M Busch, Thomas Sura, Uwe Völker

**Affiliations:** Department Functional Genomics, Interfaculty Institute of Genetics and Functional Genomics, University Medicine Greifswald, Greifswald, MV 17475, Germany; Department Functional Genomics, Interfaculty Institute of Genetics and Functional Genomics, University Medicine Greifswald, Greifswald, MV 17475, Germany; German Centre for Cardiovascular Research (DZHK), Partner Site Greifswald, Greifswald 17475, Germany; Department Functional Genomics, Interfaculty Institute of Genetics and Functional Genomics, University Medicine Greifswald, Greifswald, MV 17475, Germany; Department Functional Genomics, Interfaculty Institute of Genetics and Functional Genomics, University Medicine Greifswald, Greifswald, MV 17475, Germany; Department Functional Genomics, Interfaculty Institute of Genetics and Functional Genomics, University Medicine Greifswald, Greifswald, MV 17475, Germany; Department Functional Genomics, Interfaculty Institute of Genetics and Functional Genomics, University Medicine Greifswald, Greifswald, MV 17475, Germany; Department Functional Genomics, Interfaculty Institute of Genetics and Functional Genomics, University Medicine Greifswald, Greifswald, MV 17475, Germany; Department Functional Genomics, Interfaculty Institute of Genetics and Functional Genomics, University Medicine Greifswald, Greifswald, MV 17475, Germany; Department Functional Genomics, Interfaculty Institute of Genetics and Functional Genomics, University Medicine Greifswald, Greifswald, MV 17475, Germany; German Centre for Cardiovascular Research (DZHK), Partner Site Greifswald, Greifswald 17475, Germany

## Abstract

**Summary:**

Proteome studies frequently encounter challenges in down-stream data analysis due to limited bioinformatics resources, rapid data generation, and variations in analytical methods. To address these issues, we developed SpectroPipeR, an R package designed to streamline data analysis tasks and provide a comprehensive, standardized pipeline for Spectronaut^®^ DIA-MS data. This novel package automates various analytical processes, including XIC plots, ID rate summary, normalization, batch and covariate adjustment, relative protein quantification, multivariate analysis, and statistical analysis, while generating interactive HTML reports for e.g. ELN systems.

**Availability and implementation:**

The SpectroPipeR package (manual: https://stemicha.github.io/SpectroPipeR/) was written in R and is freely available on GitHub (https://github.com/stemicha/SpectroPipeR).

## 1 Introduction

Proteome studies are crucial for elucidating complex protein networks and functions within biological systems. Over the past decades, proteomics has evolved significantly, driven by advancements in mass spectrometry (MS) technologies (Peters-Clarke *et al.* 2024). Data-independent acquisition (DIA) has emerged as a powerful method for proteome analysis, offering advantages over data-dependent acquisition (DDA). While DDA uses a selective, intensity-driven approach to isolate and fragment the most intense precursor ions, DIA methodically fragments all ions within a predetermined mass-to-charge (*m*/*z*) range over the liquid chromatography gradient. This parallel, unbiased, and continuous measurement process generates a more comprehensive dataset, providing quantitative information that ensures high-precision, robust, and reliable quantitation (Vowinckel *et al.* 2013, Michalik *et al.* 2017, Krasny and Huang 2021).

The widespread adoption of DIA-MS on different mass spectrometers over the past decade has led to an exponential increase in research papers referencing this technique (Peters-Clarke *et al.* 2024). However, researchers frequently encounter significant challenges during down-stream data analysis, primarily due to high bioinformatics demands, rapid raw data generation, and variations in MS analysis methods. Spectronaut^®^ (Bruderer *et al.* 2015) and DIA-NN (Demichev *et al.* 2020) have emerged as key tools for DIA-MS raw data analysis and generation of quantitative ion data, compatible with a range of MS devices. They have gained extensive recognition and acceptance within the research community.

Subsequently, to the raw data analysis, the down-stream data analysis presents additional challenges, particularly in terms of computational intensity and specialized bioinformatics expertise requirements. To address these issues, various computational tools and pipelines have been developed, with R being a popular environment in the scientific community for proteomics data analysis. Although Spectronaut^®^ includes basic down-stream analysis features such as plots and tables, these outputs often require substantial modifications to meet publication standards. Thus, a downstream analysis solution, such as an R package, would be advantageous and more flexible. Despite the availability of numerous R packages and pipelines such as DEqMS (Zhu *et al.* 2020), prolfqua (Wolski *et al.* 2023), iq (Pham *et al.* 2020), MSstats (Choi *et al.* 2014), and FragPipeAnalystR (Hsiao *et al.* 2024) designed to streamline label-free quantification (LFQ) proteome data analysis, these tools often require significant pre-existing knowledge and informatics skills. These demands can pose a significant barrier to many researchers, especially those lacking advanced computational skills. To address these challenges, we developed SpectroPipeR, an R package that simplifies data analysis tasks, reduces scientists’ workload, and provides standardized outputs and reports for Spectronaut^®^ down-stream data analysis in core facilities.

## 2 Approach and implementation

SpectroPipeR is a package developed in the programming language R and has been designed to address the challenges faced in proteome studies focusing on label-free quantitation based on DIA-MS data. The package is compatible with R version ≥4.0 and Spectronaut^®^ version ≥18.7.24056. The comprehensive SpectroPipeR pipeline provides a fully automated and standardized data analysis, offering a solution for bottlenecks often encountered in proteomics research. The development of SpectroPipeR was driven by the need to simplify data analysis tasks, reduce the workload for scientists, and offer a user-friendly, scalable platform that produces uniform outputs manifested as graphs, tables, and reports in a convenient folder structure.

**Figure 1.**
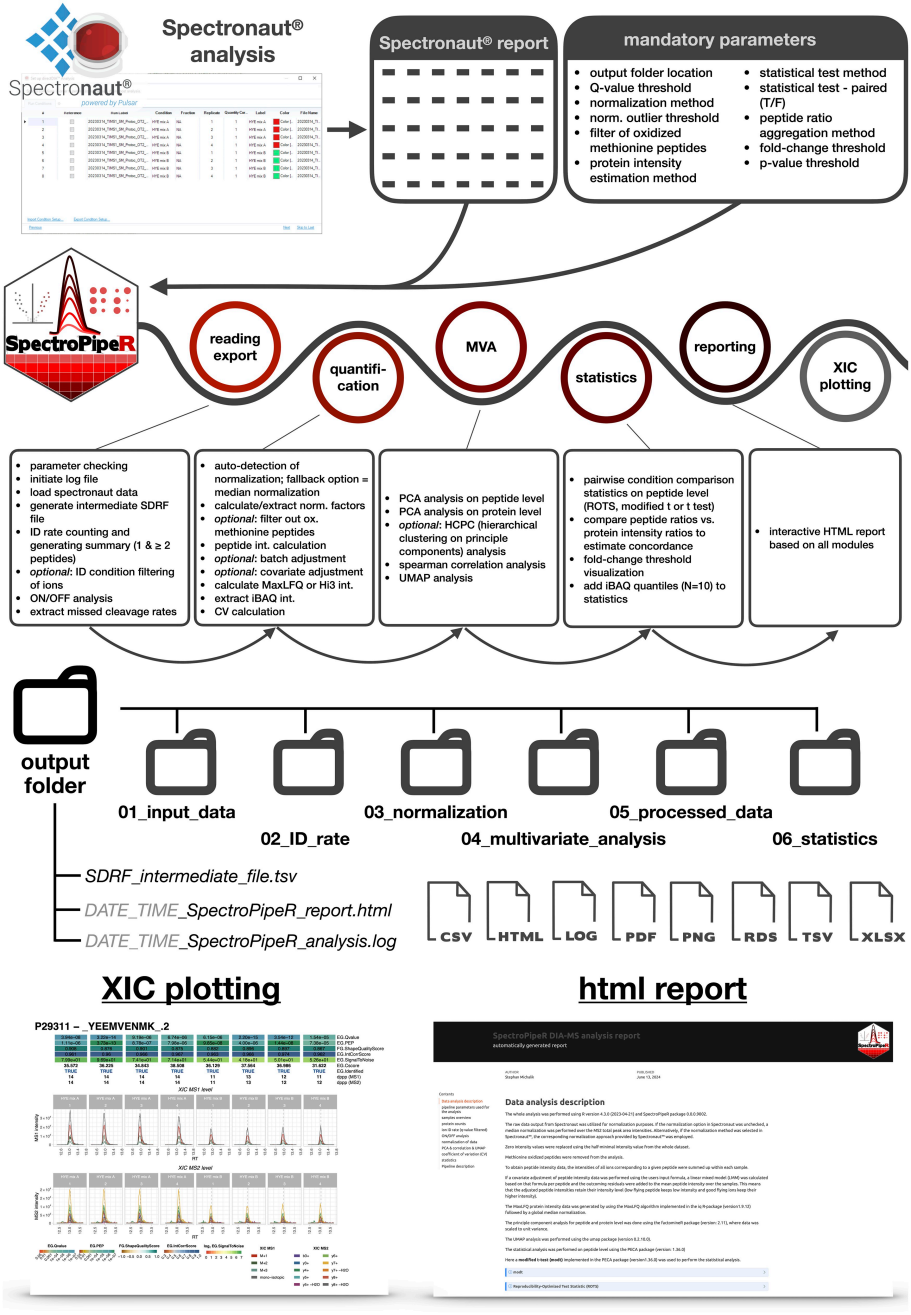
The figure summarizes the workflow and functionalities of SpectroPipeR providing a visual representation of the export folder structure, reports, and XIC plots, thereby offering a comprehensive overview of the package operations and selected output (reading report).

The functionality of SpectroPipeR encompasses a wide range of data analysis tasks, making it a versatile tool for proteomic researchers. It facilitates XIC (extracted ion chromatogram) plotting, ID rate summary, ON/OFF analysis, data normalization, batch or covariate adjustment of peptide intensities, protein quantification (iBAQ, Hi3, and MaxLFQ), multivariate analysis, peptide-centric statistical analysis (ROPECA, modified *t*-test, *t*-test) and standalone-html interactive report generation (Fig. 1). Optional parameters, such as the removal of oxidized methionine peptides and condition-wise filtering, significantly enhance the functionality of SpectroPipeR. These features allow for more precise and tailored analyses (Supplementary Figs S3–S9), accommodating the diverse needs of proteomics researchers.

The default statistical framework of the pipeline uses the peptide-centric ROPECA method, which is renowned for its minimal rate of false positives (Suomi and Elo 2017).

Beyond the standard statistical outputs such as log_2_-ratios (colored), statistical scores, *P*-values, adjusted *P*-values, and effect sizes, the iBAQ intensity quantiles (Schwanhäusser *et al.* 2011) of both conditions of a comparison is colored in the user-friendly Excel table outputs (example outputs can be found under: https://github.com/stemicha/SpectroPipeR_examples; tables are described in detail in the manual: https://stemicha.github.io/SpectroPipeR/). This is achieved by using the ten iBAQ quantiles per condition as a scale for the protein abundance, which is then combined with a 2D color code. This approach provides a robust data foundation to assess the reliability of the log_2_-ratios. For instance, proteins with low abundance tend to exhibit a more divergent fold-change compared to high abundant proteins. However, this divergence can be attributed to the limited dynamic range of the mass detector. This phenomenon is particularly noticeable in species mix experiments (Navarro *et al.* 2016). The ability to discern such nuances underscores the value of the iBAQ intensities and quantiles in enhancing the user’s understanding of the data, thereby facilitating more accurate and reliable interpretations.

The architecture of SpectroPipeR is built on a modular approach (Fig. 1, Supplementary Fig. S1), consisting of a global parameter setting and four analysis modules, along with a reporting module. These modules are executed sequentially, allowing for flexibility in the analysis process. Researchers can run specific analyses independently or as part of the complete pipeline, depending on their requirements. This modular structure enhances the tool’s adaptability to various research needs. An all-in-one function that executes all modules and saves time and code lines for the user is also included, making the analysis highly convenient for new R users.

## 3 Examples of SpectroPipeR usage

In order to illustrate the analytical capabilities and report generation of the SpectroPipeR package, we used species mix data from the study conducted by Reder *et al.* (2024). Furthermore, to showcase a clinically pertinent analysis using the SpectroPipeR package, we drew upon a case-control cancer cohort study previously published by Vitko *et al.* (2024). This study includes 20 samples each from lung cancer patients and control subjects. The plasma samples were enriched using the SEER technology (NP2). The reports and results of the analysis are available at the GitHub https://github.com/stemicha/SpectroPipeR_examples. Moreover, the Supplementary Material (Supplementary Section S7) encompasses valuable code snippets tailored for SpectroPipeR.

Looking ahead, future development of SpectroPipeR may include expanding the range of supported data formats, integrating additional statistical analysis methods for time series analysis, and developing further tutorials and documentation to assist new users. These planned improvements aim to make SpectroPipeR an even more powerful and user-friendly tool for proteome data analysis, ultimately advancing the field of proteomics.

## 4 Conclusion

SpectroPipeR represents a significant advancement in proteomics data analysis tools. By addressing the challenges of limited bioinformatics resources, rapid data generation, and variations in analysis methods, it streamlines the data analysis process and ensures high-quality, reproducible results. As proteomics continues to play a crucial role in understanding complex biological systems, tools like SpectroPipeR will be instrumental in accelerating research and discovery in this field. Its ability to simplify complex analyses, provide standardized outputs, and generate publication-ready results positions SpectroPipeR as a valuable asset for proteomics researchers.

## Supplementary Material

btaf086_Supplementary_Data

## Data Availability

The data and resources used in this study are accessible through the following links: SpectroPipeR manual available at: https://stemicha.github.io/SpectroPipeR/. SpectroPipeR examples can be found at: https://github.com/stemicha/SpectroPipeR_examples. For SpectroPipeR example datasets and scripts, refer to: https://doi.org/10.5281/zenodo.14849402. Additional supplemental material for SpectroPipeR is available at *Bioinformatics* online.
