# Supplementary material for: SpectroPipeR—a streamlining post Spectronaut® DIA-MS data analysis R package
Source: Bioinformatics. 2025 Feb 22;41(3):btaf086. doi: 10.1093/bioinformatics/btaf086 (PMC11893148; doi:10.1093/bioinformatics/btaf086)
Supplement: btaf086_Supplementary_Data [file btaf086_supplementary_data.pdf]

SpectroPipeR - a streamlining post Spectronaut® DIA-MS data analysis R package - supplemental material

Table of contents

1 SpectroPipeR data analysis 2

2 online resources for SpectroPipeR 2

3 example usage of SpectroPipeR 3
3.1 introduction . . . 3
3.2 example analysis code . . . 3
3.3 example XIC code . . . 3

4 selected optional SpectroPipeR settings 4
4.1 remove methionine oxidized peptides before quantitative analysis . . . 4
4.2 SpectroPipeR condition wise filtering . . . 9

5 differential abundant protein (DAP) benchmarking using species mix measurements 11
5.1 samples . . . 11
5.2 analysis . . . 12
5.2.1 Spectronaut® parameters . . . 12
5.2.2 analysis in SpectroPipeR . . . 12
5.2.3 analysis with Prolfqua . . . 12
5.2.4 analysis with DAP-MS (MSqRob,MS-EmpiRe,DEqMS) . . . 13
5.2.5 DAP analysis results . . . 13

6 SpectroPipeR processing time 16
6.1 Processing time example . . . 16
6.1.1 Results of the processing time example . . . 17

7 Code snippets for SpectroPipeR 18
7.1 installation of SpectroPipeR . . . 18
7.2 simple SpectroPipeR analysis . . . 18
7.3 SpectroPipeR XIC plots . . . 18
7.4 SpectroPipeR gui . . . 19

References 20

1 SpectroPipeR data analysis

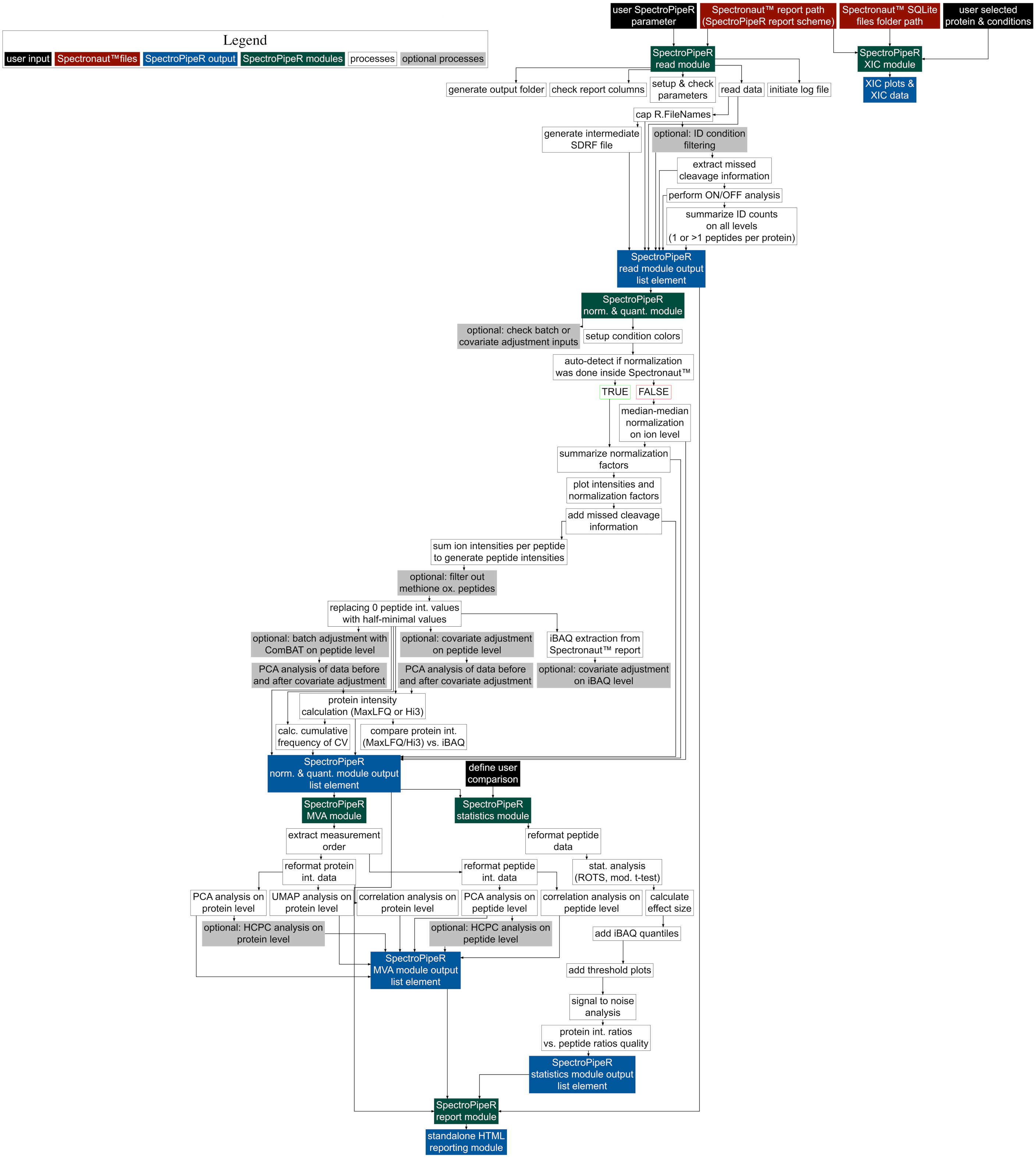

suppl. figure 1. *SpectroPipeR data analysis flow chart (simplified)*

2 online resources for SpectroPipeR

- SpectroPipeR package: https://github.com/stemicha/SpectroPipeR

- SpectroPipeR manual: https://stemicha.github.io/SpectroPipeR/
- examples of SpectroPipeR data analysis: https://github.com/stemicha/SpectroPipeR_examples
- example of SpectroPipeR data analysis HTML reports: https://stemicha.github.io/SpectroPipeR_examples/

3 example usage of SpectroPipeR

3.1 introduction

In the realm of mass spectrometry, species mix experiments have become a standard procedure, routinely employed in core facilities worldwide. These experiments are used to benchmark the performance of various methods and devices. For the analysis we used the Exploris480 data published by Reder *et al.* (2023).

The data can be obtained from:

- repository: https://massive.ucsd.edu/ProteoSAFe/static/massive.jsp
- repository ID: MSV000092489

The analysis with SpectroPipeR below was performed using default parameters.

3.2 example analysis code

```
# load SpectroPipeR
library(SpectroPipeR)

# SpectroPipeR analysis in one function using default parameter
SpectroPipeR_analysis <- SpectroPipeR(
  file = "HYE_Exploris480_SN19_Report_SpectroPipeR (Normal).tsv",
  parameter = list(output_folder = "species_mix_analysis"),
  condition_comparisons = cbind(c("HYE mix A","HYE mix B"))
)
```

The detailed analysis tables, plots and report can be found at

https://github.com/stemicha/SpectroPipeR_examples.

3.3 example XIC code

As an example, the Cold shock protein CspA - P0A9X9 was choosen (FC: -4.248; q-value: 1.685e-07), which should exhibit an abs. fold-change of 4. An examplary XIC plot of one ion is shown in suppl. figure 2.

```
# load SpectroPipeR
library(SpectroPipeR)

XIC_plot_module(
  Spectronaut_report_path = "HYE_Exploris480_SN19_Report_SpectroPipeR (Normal).tsv",
  Spectronaut_xicDB_path = "HYE_Exploris480_SN19_XIC-DBs",
  protein_groups = "P0A9X9",
  number_of_cores = 2,
  output_path = "species_mix_analysis/XIC_plots",
  export_csv_files = F
  )
```

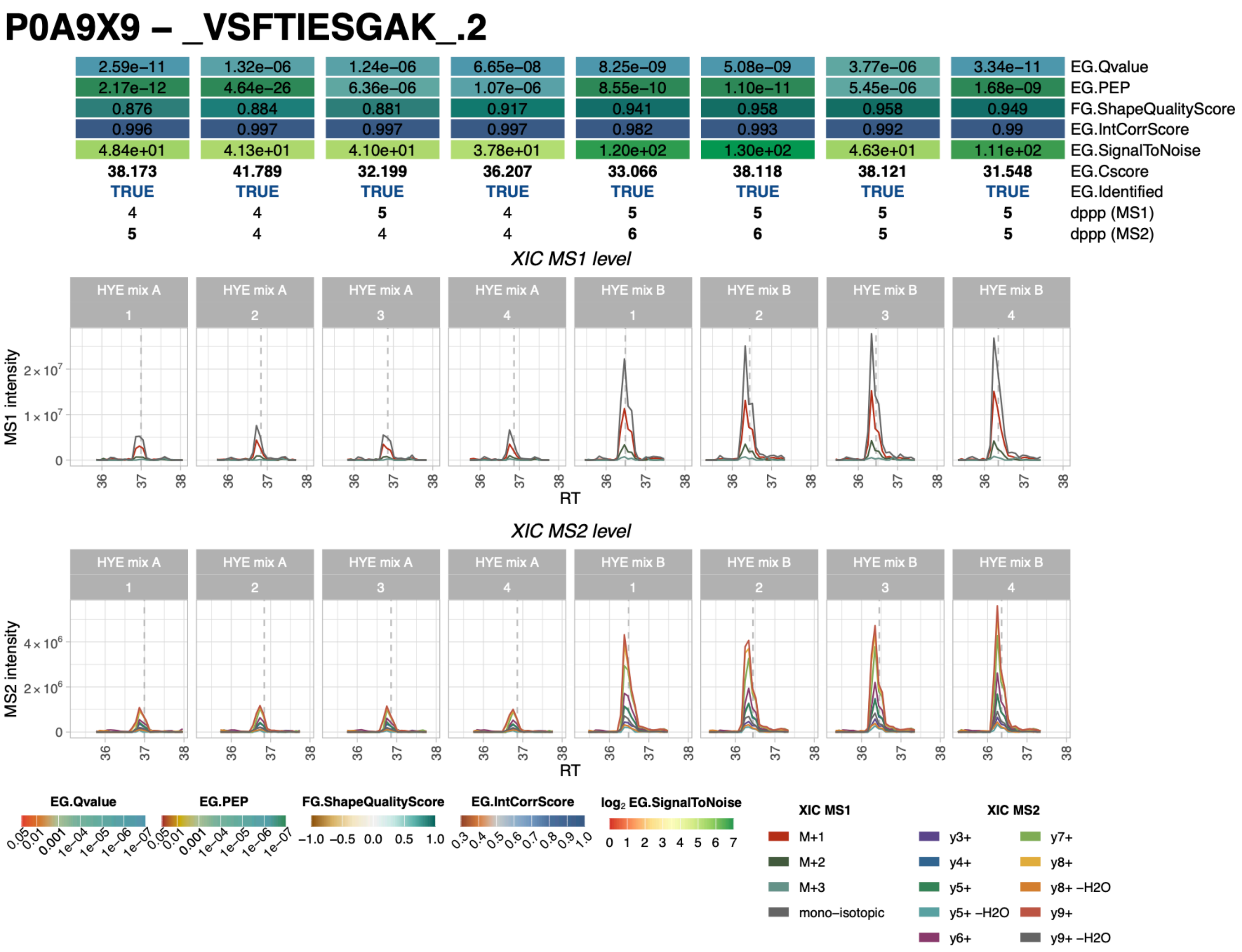

suppl. figure 2. *VSFTIESGAK +2 ion of the protein CspA (P0A9X9)*

Other XICs of ions of the protein CspA (P0A9X9) of the analysis can be found at

https://github.com/stemicha/SpectroPipeR_examples/tree/main/species_mix_analysis/XIC_plots.

4 selected optional SpectroPipeR settings

4.1 remove methionine oxidized peptides before quantitative analysis

Methionine oxidation during sample preparation is a significant concern, particularly when using e.g. 2-chloroacetamide (CA) as an alkylating agent. The oxidation of methionine increases dramatically with CA, affecting up to 40% of all Met-containing peptides, compared to only 2-5% with iodoacetamide (IOA) (Hains and Robinson (2017)). This extensive oxidation can cause qualitative and quantitative issues in large-scale proteomics studies, complicating data analysis and increasing the search space required for database matching.

Methionine oxidation has been also observed to accumulate spuriously during the initial stages of a typical bottom-up proteomics workflow. Notably, the extent of methionine oxidation increases with prolonged trypsin digestion and higher ionization energy during electrospray ionization (ESI) (Zang *et al.* (2012); Chen and Cook (2007)).

These observations complicate the differentiation between methionines oxidized *in vivo* and those artifactually oxidized *in vitro* during sample preparation and mass spectrometric analysis.

SpectroPipeR includes an option to remove oxidized methionine peptides.

```
# SpectroPipeR analysis with removing oxidized methionine peptides
SpectroPipeR_analysis <- SpectroPipeR(file = "Spectronaut_SpectroPipeR_report_file.tsv",
                                      parameter = list(output_folder = "output_folder",
                                                       # remove Met-ox. peptides
                                                       filter_oxidized_peptides = TRUE),
                                      condition_comparisons = cbind(c("HYE mix A",
                                                                      "HYE mix B"))
                                      )
```

We employed the HYE (human-yeast-*E.coli*) species mix, analyzed using an UltiMate 3000 system coupled with an Exploris™ 480 mass spectrometer (data set from Reder *et al.* (2023)), to exemplify the methionine oxidized and non-oxidized counterpart ions. The suppl. figure 3 displays the mix composition.

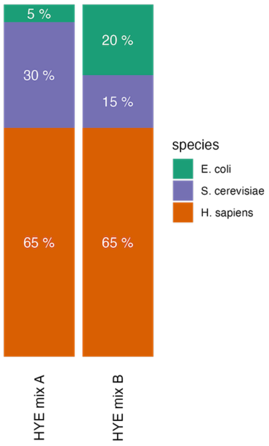

suppl. figure 3. *HYE species mix composition*

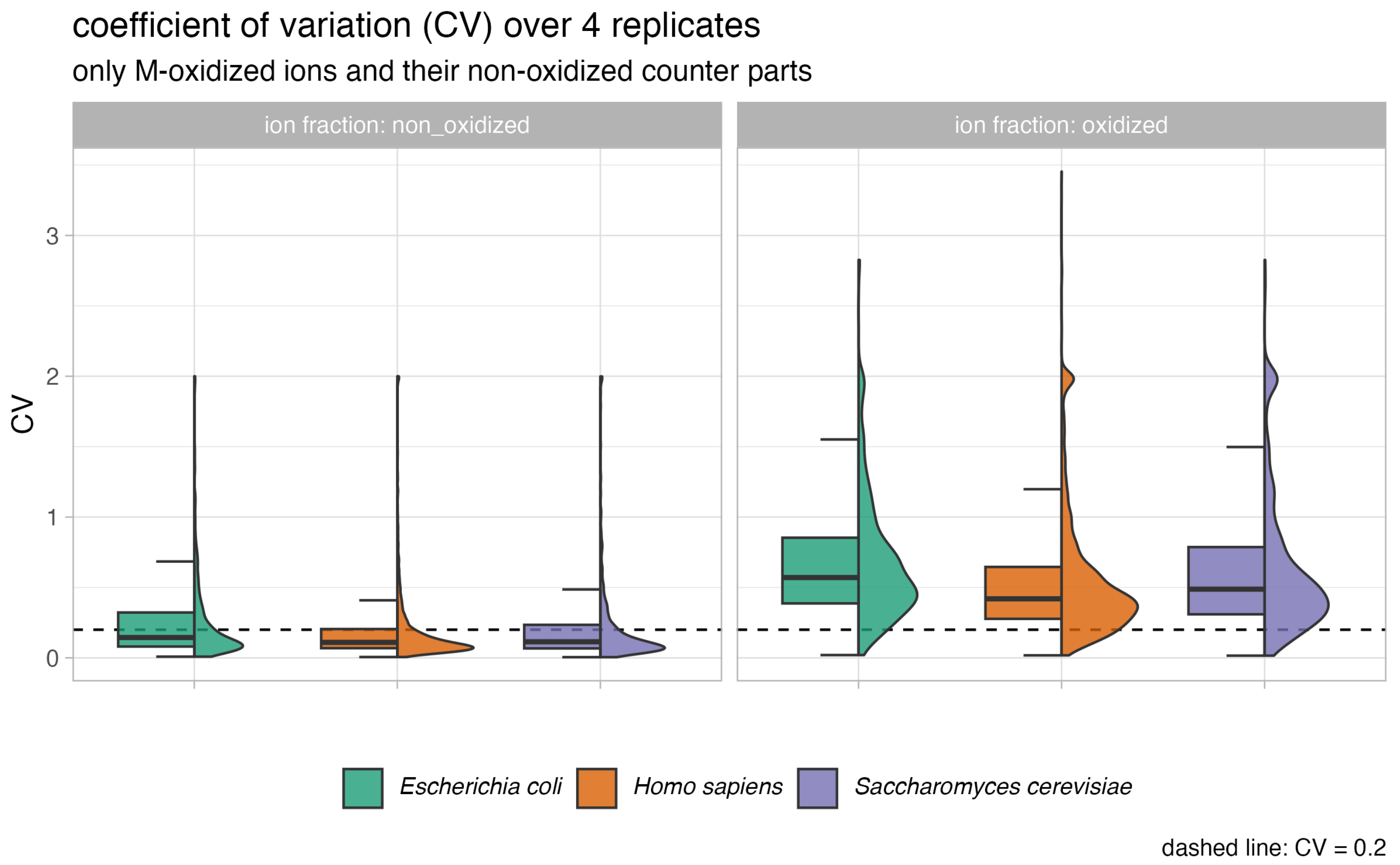

suppl. figure 4. *HYE species mix results: coefficient of variation plot of ion ratios (HYE mix A / HYE mix B) containing oxidized methionines or their unmodified forms*

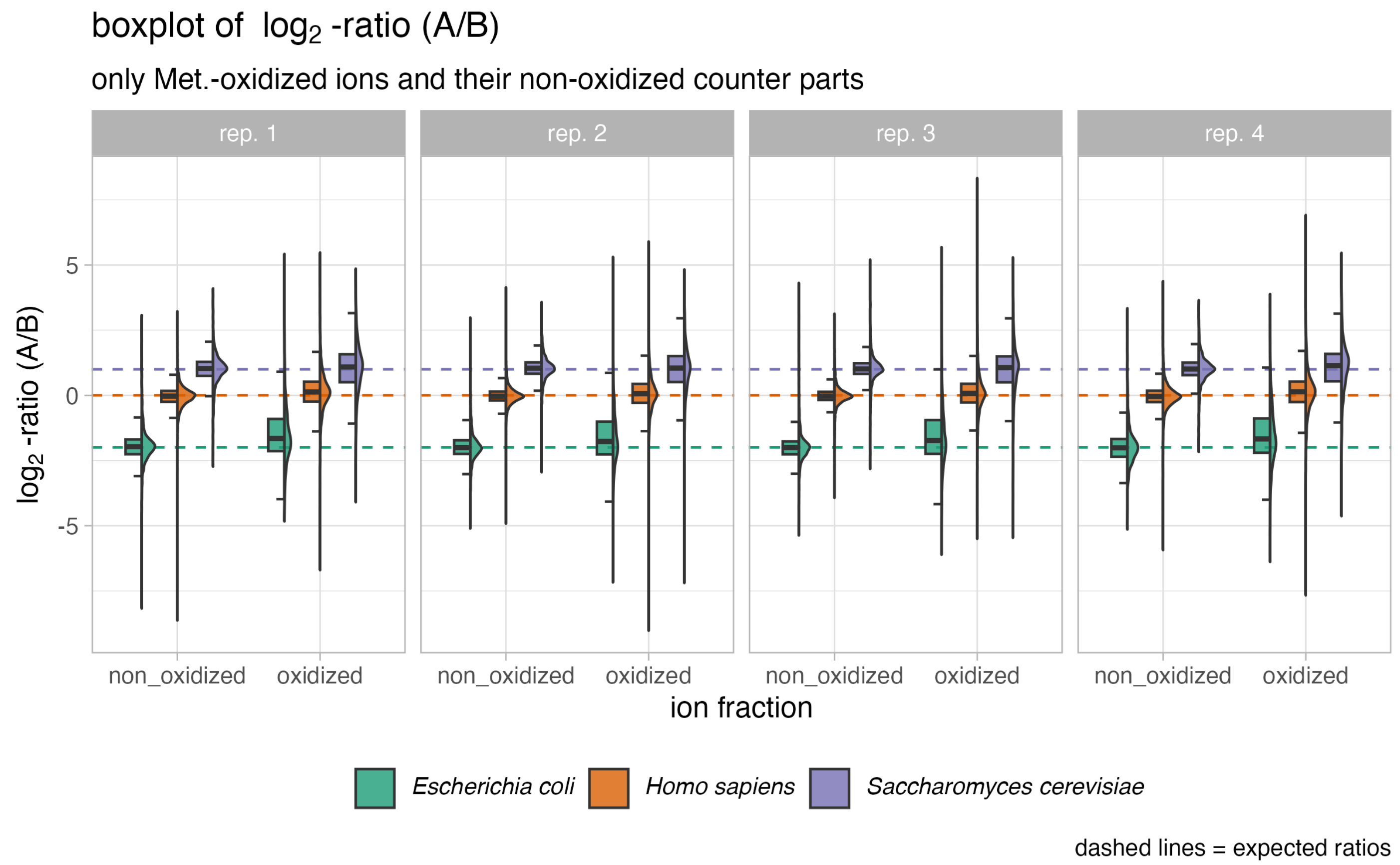

suppl. figure 5. *HYE species mix results: ratios (A/B) boxplot of ions containing oxidized methionines and their unmodified forms. The dashed lines diplay the expected ratio per species.*

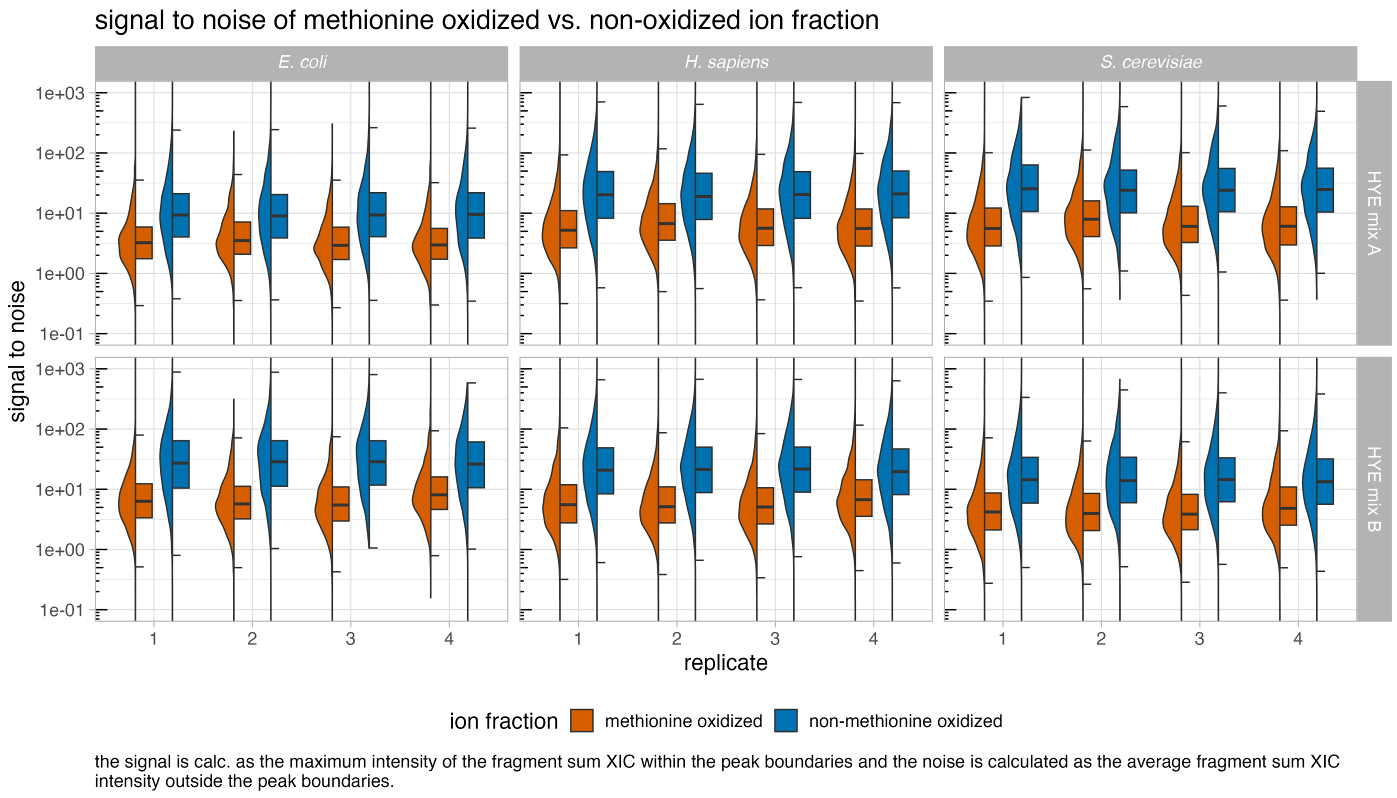

suppl. figure 6. *signal to noise of the non-oxidized and methionine-oxidized ion fraction.*

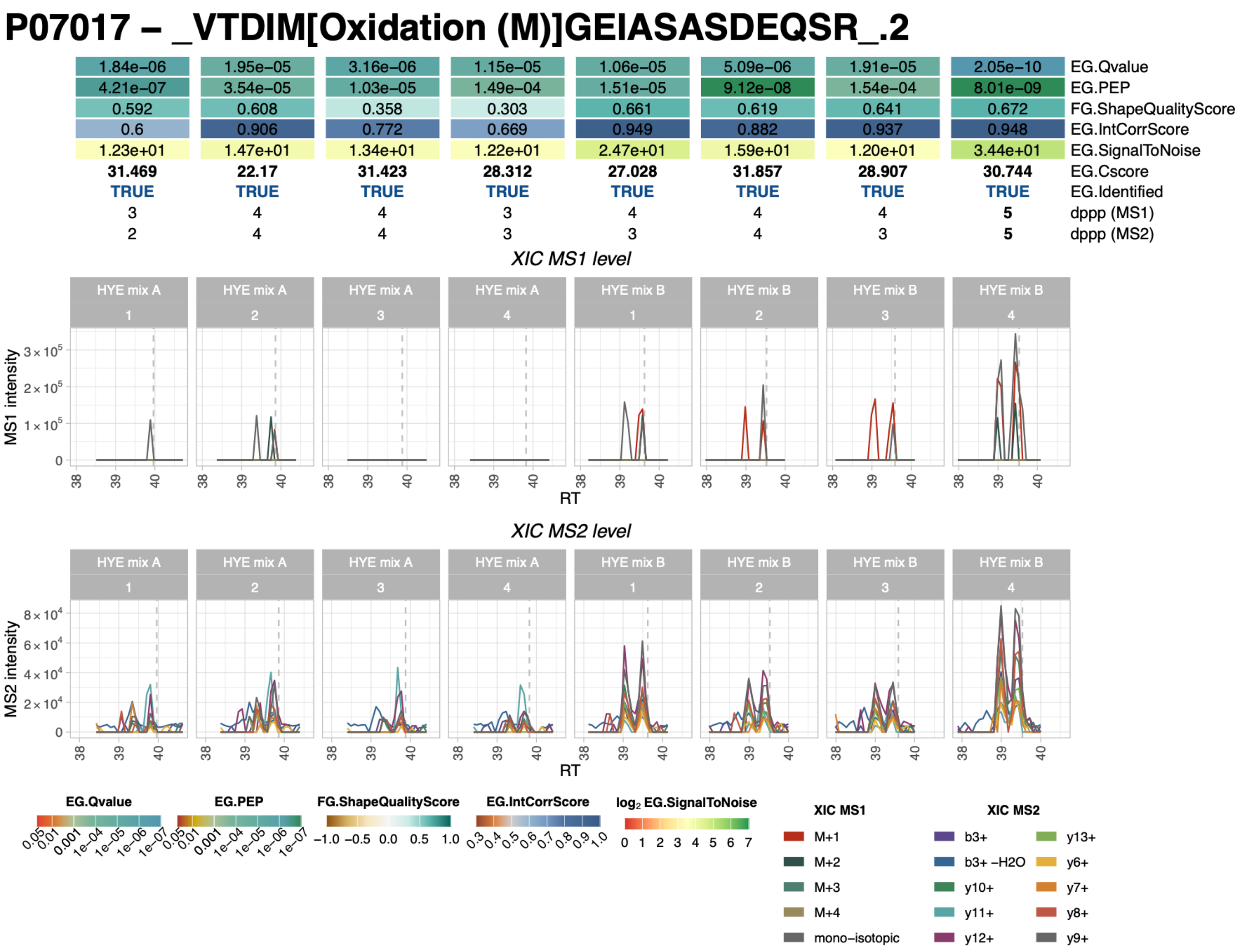

suppl. figure 7. *The XIC plot diplays the methionine oxidized fraction of the VTDIMGEIASASDEQSR+2 ion.*

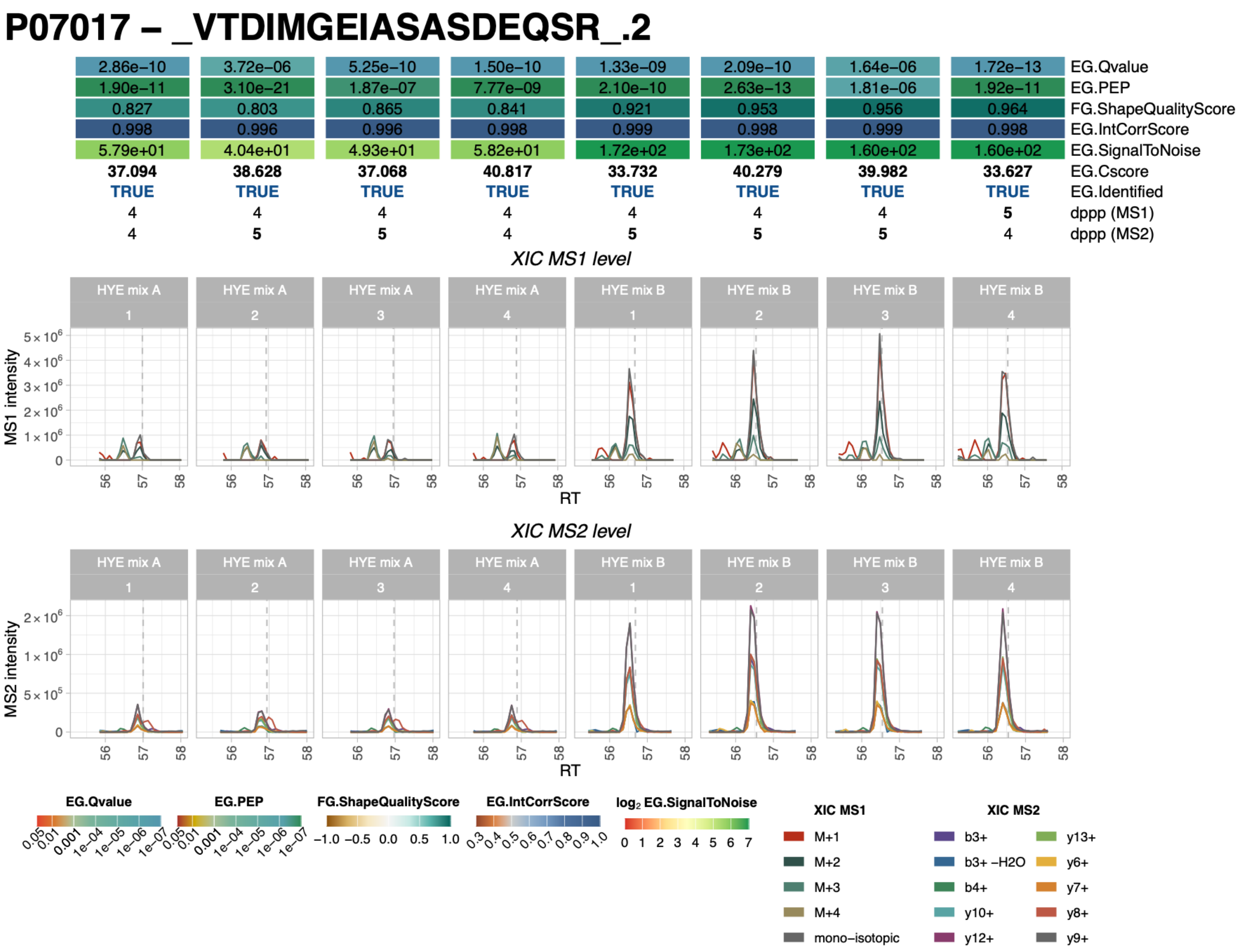

suppl. figure 8. *The XIC plot diplays the un-modified fraction of the VTDIMGEIASASDEQSR+2 ion.*

The analysis of species mix samples revealed a increasing coefficient of variation for ions containing methionine oxidation compared to their un-oxidized counterparts (suppl. figure 4). This trend is also evident in the ratio boxplots (suppl. figure 5), where the oxidized methionine fraction of ions exhibited significantly higher variation from the expected ratios across all four replicates compared to the un-oxidized ions. Additionally, the signal-to-noise ratio (suppl. figure 6) for the oxidized methionine ion fraction was lower than that of the non-oxidized fraction, resulting in better detectability and less variation in the quantification of the non-oxidized ions. An exemplary ion (VTDIMGEIASADEQSR+2; P07017; Methyl-accepting chemotaxis protein II; Tar) from *E. coli* with an expected ratio of 4 is shown in suppl. figure 7 (methionine oxidized ion) and suppl. figure 8 (unmodified ion). This example clearly demonstrates that oxidized ions are less intense, have a poorer signal-to-noise ratio, and consequently exhibit a worse coefficient of variation, failing to match the expected fold-change of the HYE species mix.

In summary, it is recommended in SpectroPipeR (not mandatory) to exclude methionine-oxidized ions, as the species mix experiments have shown that the non-oxidized (methionine-containing peptides) fraction exhibits a lower coefficient of variation, generally better signal-to-noise ratio, and better conservation of expected ratios compared to the oxidized fraction of the peptides.

4.2 SpectroPipeR condition wise filtering

The `ID_condition_filtering` option, when used in conjunction with `ID_condition_filtering_percent`, enables users to filter ions that are present in a specified proportion of replicates per condition. This functionality facilitates the exclusion of ions that are, for instance, detected only once within a given condition.

```
# SpectroPipeR analysis with ions only present in 100% of replicates per condition
SpectroPipeR_analysis <- SpectroPipeR(file = example_file_path,
                                      parameter = params,
```

```r
                                             ID_condition_filtering = T,
                                             ID_condition_filtering_percent = 1,
                                             condition_comparisons = cbind(c("HYE mix A",
                                                                             "HYE mix B"))
                                             )
```

```r
# SpectroPipeR analysis with ions only present in 50% of replicates per condition
SpectroPipeR_analysis <- SpectroPipeR(file = "Spectronaut_SpectroPipeR_report_file.tsv",
                                             parameter = list(output_folder = "output_folder"),
                                             ID_condition_filtering = T,
                                             ID_condition_filtering_percent = 0.5,
                                             condition_comparisons = cbind(c("HYE mix A",
                                                                             "HYE mix B"))
                                             )
```

The following schematic (suppl. figure 9) illustrates the condition-specific filtering process in SpectroPipeR.

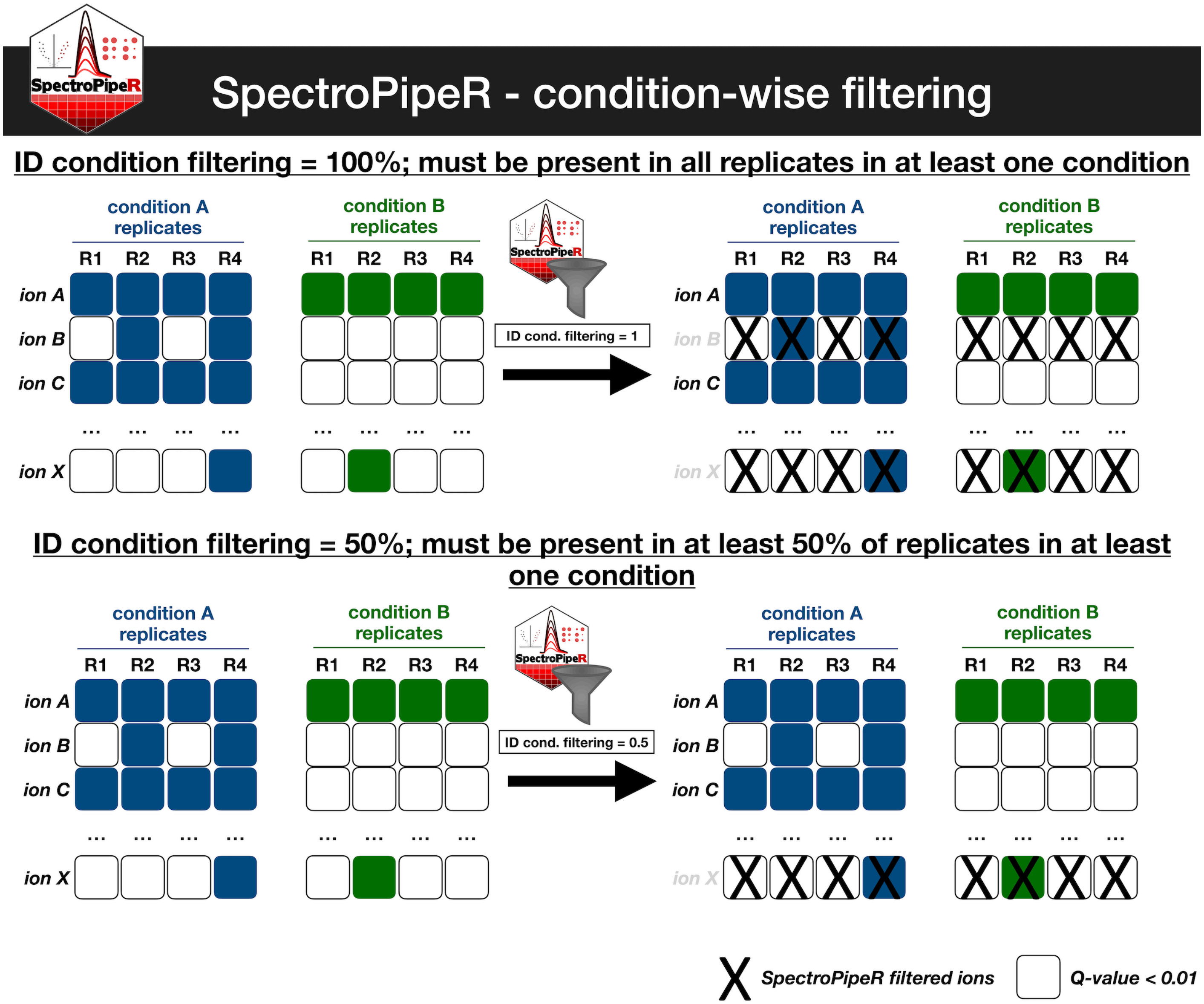

suppl. figure 9. *SpectroPipeR condition wise filtering*

The additional filtering step can potentially enhance the robustness of your data by ensuring that only ions detected across multiple replicates are utilized for quantitative and statistical analysis.

5 differential abundant protein (DAP) benchmarking using species mix measurements

To evalulate the results of SpectroPipeR and other tools we used the metric of DAP counts (differential abundant protein counts) in HYE (human-yeast-*E. coli*) species mix experiments. Therefore, the statistics tables of the analyses were employed to enumerate the differentially abundant proteins (DAP), utilizing various adjusted p-values and species-specific fold-change thresholds. Alternatively, only adjusted p-values were used for filtering DAP proteins. For *E. coli*, the applied fold-change threshold was < -3 (expected: -4), whereas for *S. cerevisiae*, it was > 1.5 (expected: 2)(suppl. figure 10). Since the *H. sapiens* proteome fraction was used for the normalization of samples, the DAP counting was omitted for this fraction.

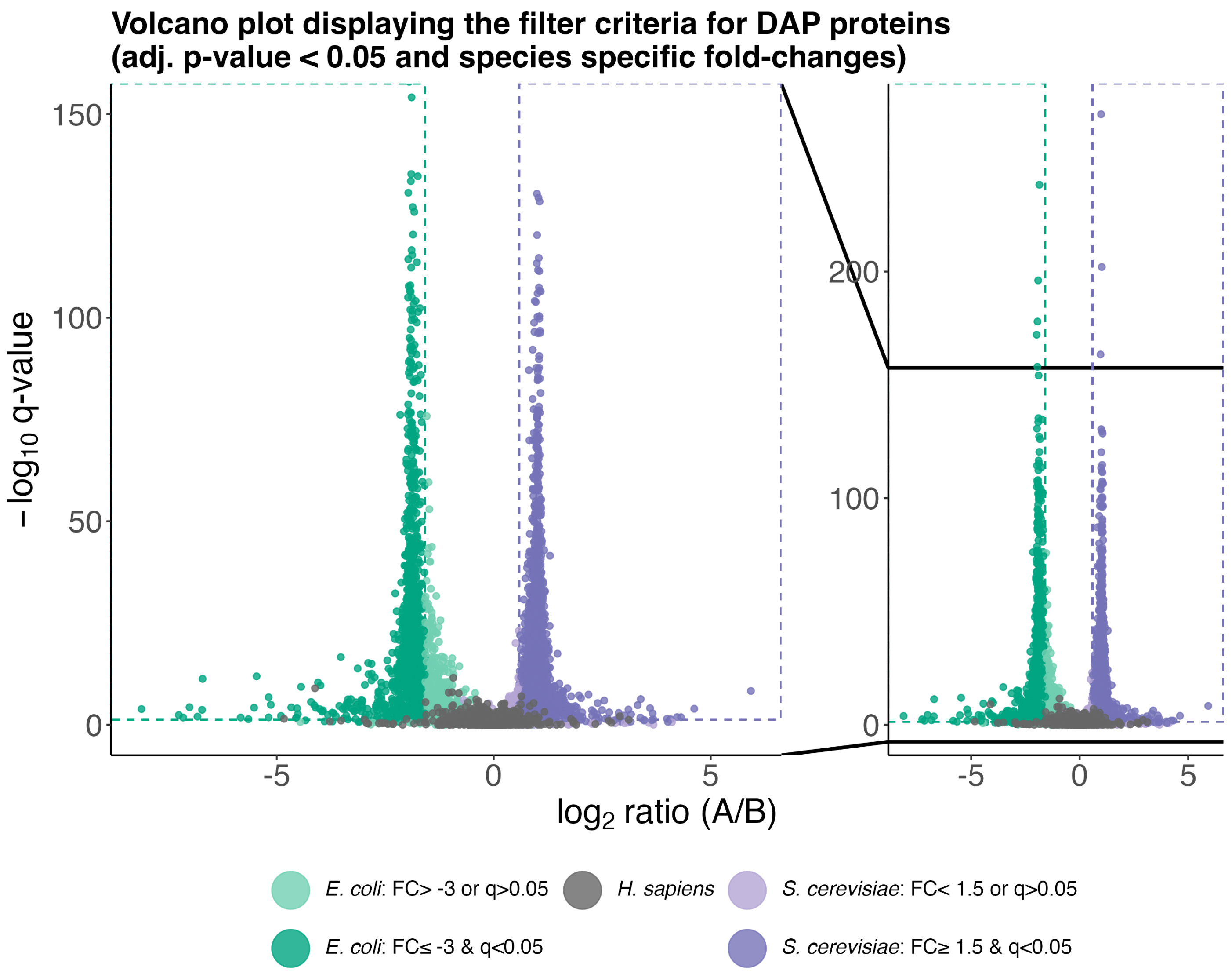

suppl. figure 10. *Exemplary plot volcano-plot with the log2 HYE species mix ratios (A/B) x-axis and the negative decadic logarithm of the q-value (adjusted p-value) of the statistical analysis on the y-axis is displayed. Greenish colors represent* E. coli *proteins, grey mark human proteins and purple dots display* S. cerevisiae *proteins, with more saturated colors corresponding to the filter criteria for an affirmative quantification reading. That is, for* E. coli*, the fold-change had to be below -3 (expected value -4) together with a q-value below 0.05; and for* S. cerevisiae*, the fold-change had to be above 1.5 (expected value 2) together with a q-value below 0.05.*

5.1 samples

For the analysis we used the Exploris480 data published by Reder *et al.* (2023).

The data can be obtained from:

- repository: https://massive.ucsd.edu/ProteoSAFe/static/massive.jsp
- repository ID: MSV000092489

5.2 analysis

The data was analyzed using Spectronaut® version 19.0.240606.62635.

5.2.1 Spectronaut® parameters

- directDIA Workflow: directDIA+ (Deep)
- Pulsar Search: Enzymes / Cleavage Rules: Trypsin/P
- Pulsar Search: Min Peptide Length: 7
- Pulsar Search: Max Peptide Length: 52
- Pulsar Search: Missed Cleavages: 2
- Pulsar Search: Fixed Modifications:: Carbamidomethyl (C)
- Pulsar Search: Variable Modifications: : Oxidation (M)
- Pulsar Search: Max Variable Modifications: 5
- Pulsar Search: PSM FDR: 0.01
- Pulsar Search: Peptide FDR: 0.01
- Pulsar Search: Protein Group FDR: 0.01
- DIA Analysis: Interference Correction: True
- DIA Analysis: Cross-Run Normalization: True
- DIA Analysis: Normalization Filter Type: FASTA Name Filter (FASTA Name: Homo_sapiens)
- DIA Analysis: Precursor Qvalue Cutoff: 0.01
- DIA Analysis: Precursor PEP Cutoff: 0.2
- DIA Analysis: Protein Qvalue Cutoff (Experiment): 0.01
- DIA Analysis: Protein Qvalue Cutoff (Run): 0.05
- DIA Analysis: Protein PEP Cutoff: 0.75

5.2.2 analysis in SpectroPipeR

5.2.2.1 SpectroPipeR various settings

The settings for proteomic experiment data analysis are depend on the samples and the experimental design. For a general overview, we employed parameter settings in SpectroPipeR, which were applied to a HYE (human-yeast-*E. coli*) species mix measured using an UltiMate 3000 coupled to an Exploris™ 480 mass spectrometer setup (data set from Reder *et al.* (2023)).

Table 1: various parameter settings used for SpectroPipeR

parameter setting	condition-wise filt.	remove Met-ox. peptides	stat. test method	peptide ratio agg. alg.	protein int. alg.
A	ON using 1.0	ON	modt	median	MaxLFQ
B	ON using 1.0	OFF	modt	median	MaxLFQ
C	ON using 1.0	ON	rots	median	MaxLFQ
D	ON using 1.0	OFF	rots	median	MaxLFQ
E	OFF	ON	modt	median	MaxLFQ
F	ON using 0.5	ON	modt	median	MaxLFQ
G	ON using 0.5	OFF	modt	median	MaxLFQ
H	OFF	OFF	modt	median	MaxLFQ
I	OFF	ON	rots	median	MaxLFQ
J	ON using 0.5	ON	rots	median	MaxLFQ
K	OFF	OFF	rots	median	MaxLFQ
L	ON using 0.5	OFF	rots	median	MaxLFQ

5.2.2.2 SpectroPipeR settings for the comparison with other tools

The analysis in SpectroPipeR for the DAP count comparison with other tools was conducted with the removal of oxidized methionine peptides setting together with various condition filtering settings (OFF, 0.5, or 1.0) before using the modified t-test statistics.

5.2.3 analysis with Prolfqua

The Prolfqua analysis was performed with default parameters using the MaxLFQ intensities from Spectronaut® (condition filtering = OFF) or from SpectroPipeR (condition filtering = 1.0).

5.2.4 analysis with DAP-MS (MSqRob,MS-EmpiRe,DEqMS)

For the DAP-MS (Koopmans *et al.* (2022)) analysis we used the following settings.

DAP-MS analysis settings:

- min_detect = 2
- fraction_detect = 0.75
- min_quant = 3
- norm_algorithm = ‘vsn&modebetween_protein’
- rollup_algorithm = ‘maxlfq’
- dea_algorithm = c(“deqms”, “msempire”, “msqrob”)
- dea_log2foldchange_threshold = NA
- diffdetect_min_samples_observed = 1

5.2.5 DAP analysis results

5.2.5.1 DAP results: SpectroPipeR various settings

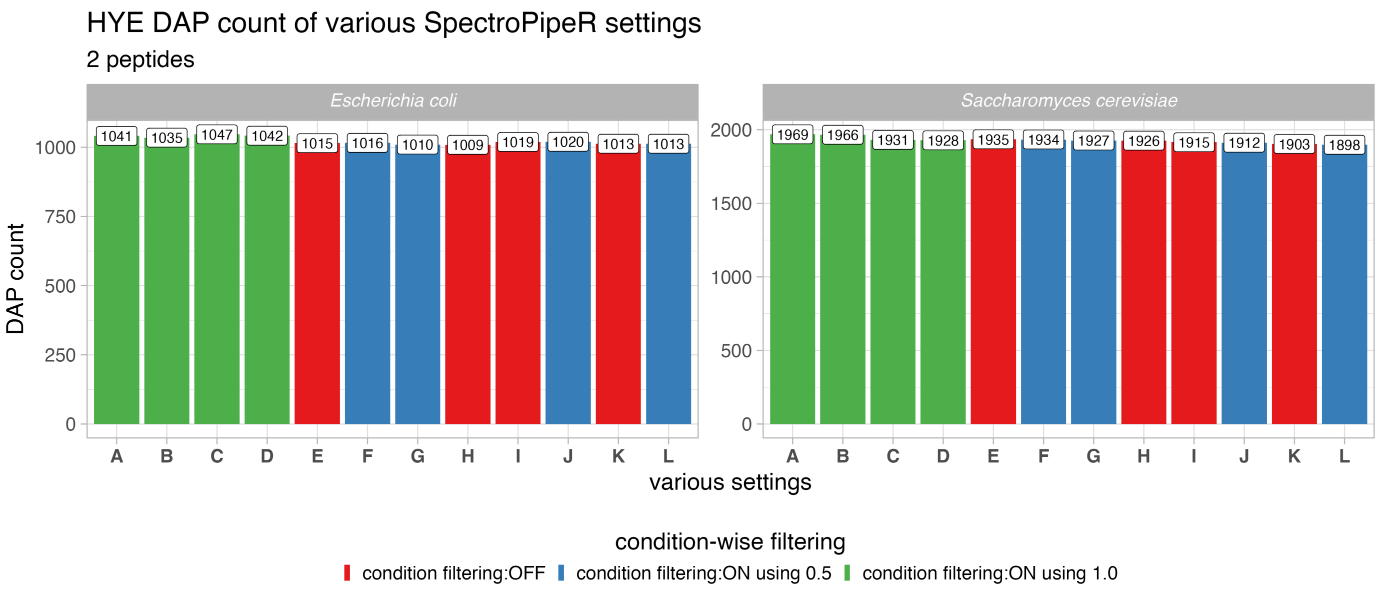

	A	B	C	D	E	F	G	H	I	J	K	L
condition-wise filtering:	ON using 1.0	ON using 1.0	ON using 1.0	ON using 1.0	OFF	ON using 0.5	ON using 0.5	OFF	OFF	ON using 0.5	OFF	ON using 0.5
remove Met-ox. peptides:	ON	OFF	ON	OFF	ON	ON	OFF	OFF	ON	ON	OFF	OFF
stat. test:	modt	modt	rots	rots	modt	modt	modt	modt	rots	rots	rots	rots
peptide ratio agg. alg.:	median	median	median	median	median	median	median	median	median	median	median	median
protein int. alg.:	MaxLFQ	MaxLFQ	MaxLFQ	MaxLFQ	MaxLFQ	MaxLFQ	MaxLFQ	MaxLFQ	MaxLFQ	MaxLFQ	MaxLFQ	MaxLFQ

suppl. figure 11. *The upper panel of the plot illustrates the count of differential abundance of proteins, filtered by adjusted p-value and absolute fold-change, for the* E. coli *and* S. cerevisiae *proteome fractions, utilizing the statistical results (proteins with at least 2 peptides) from various SpectroPipeR settings. The lower panel presents a table detailing these different settings.*

The results of the SpectroPipeR analysis tests using various settings (suppl. figure 11) were highly consistent, with the condition-wise filtering parameter exerting the greatest influence on the number of differentially abundant proteins. In this specific instance, the condition-wise filtering at 100%, the removal of oxidized methionines, peptide ratio median aggregation, and the modified t-test exhibited optimal performance. These outcomes may exhibit slight variations depending on factors such as the measurement device and the version of Spectronaut® employed.

5.2.5.2 DAP results: SpectroPipeR, DEqMS, MS-EmpiRe, MSqRob, Prolfqua

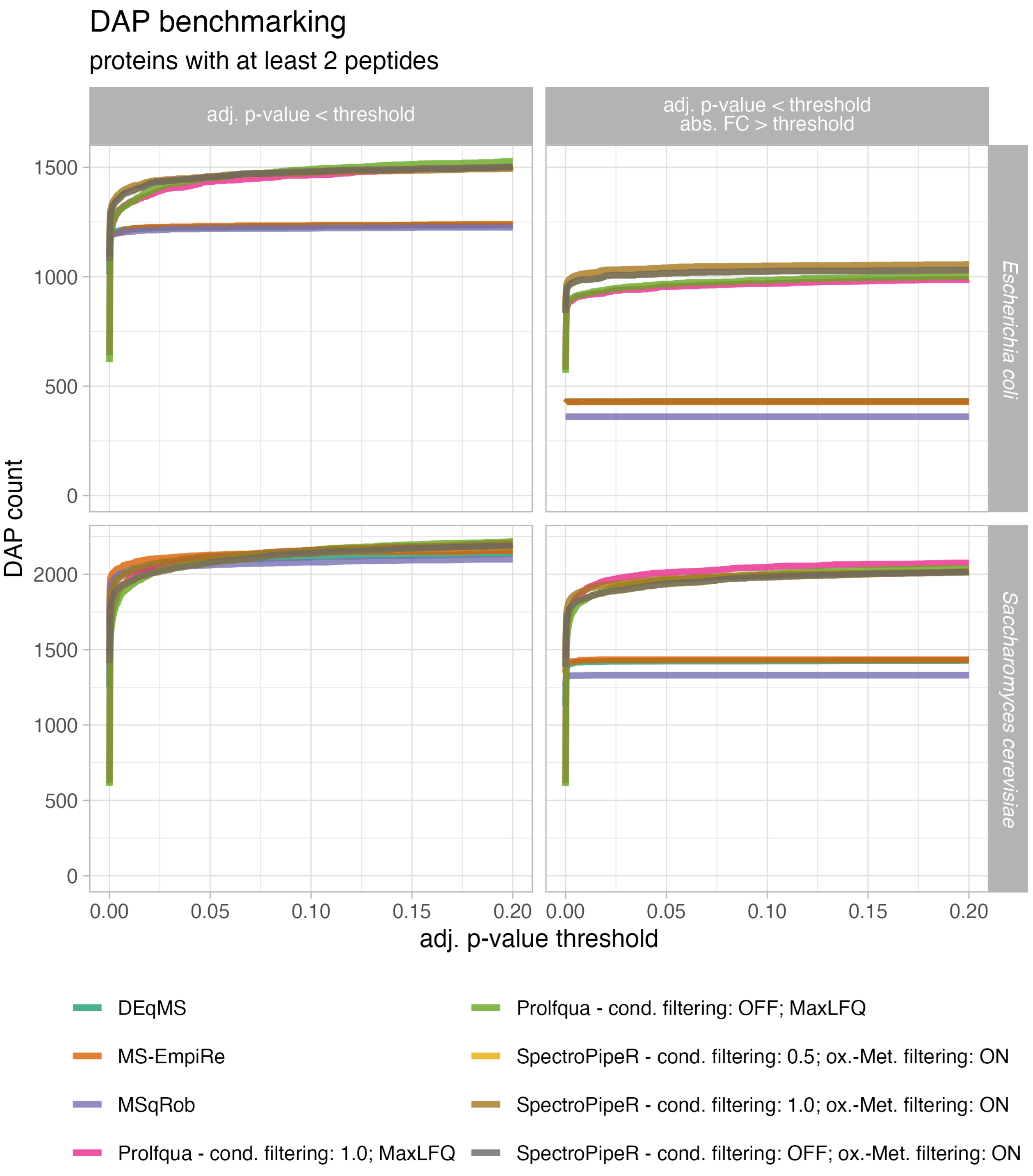

suppl. figure 12. *Benchmarking of various tools/settings was conducted using HYE species mix samples. The plot illustrates the differentially abundant proteins (DAP) based on the statistical results of the different tools and settings. The plots are categorized by species. The left panel illustrates filtering based solely on adjusted p-values, while the right panel incorporates both adjusted p-values and the species specific fold-change threshold (fold-change:* E.coli *= -3;* S. cerevisiae *= 1.5) for the differentially abundant proteins counting (DAP). The x-axis represents the adjusted p-value, and the y-axis quantifies the DAP count.*

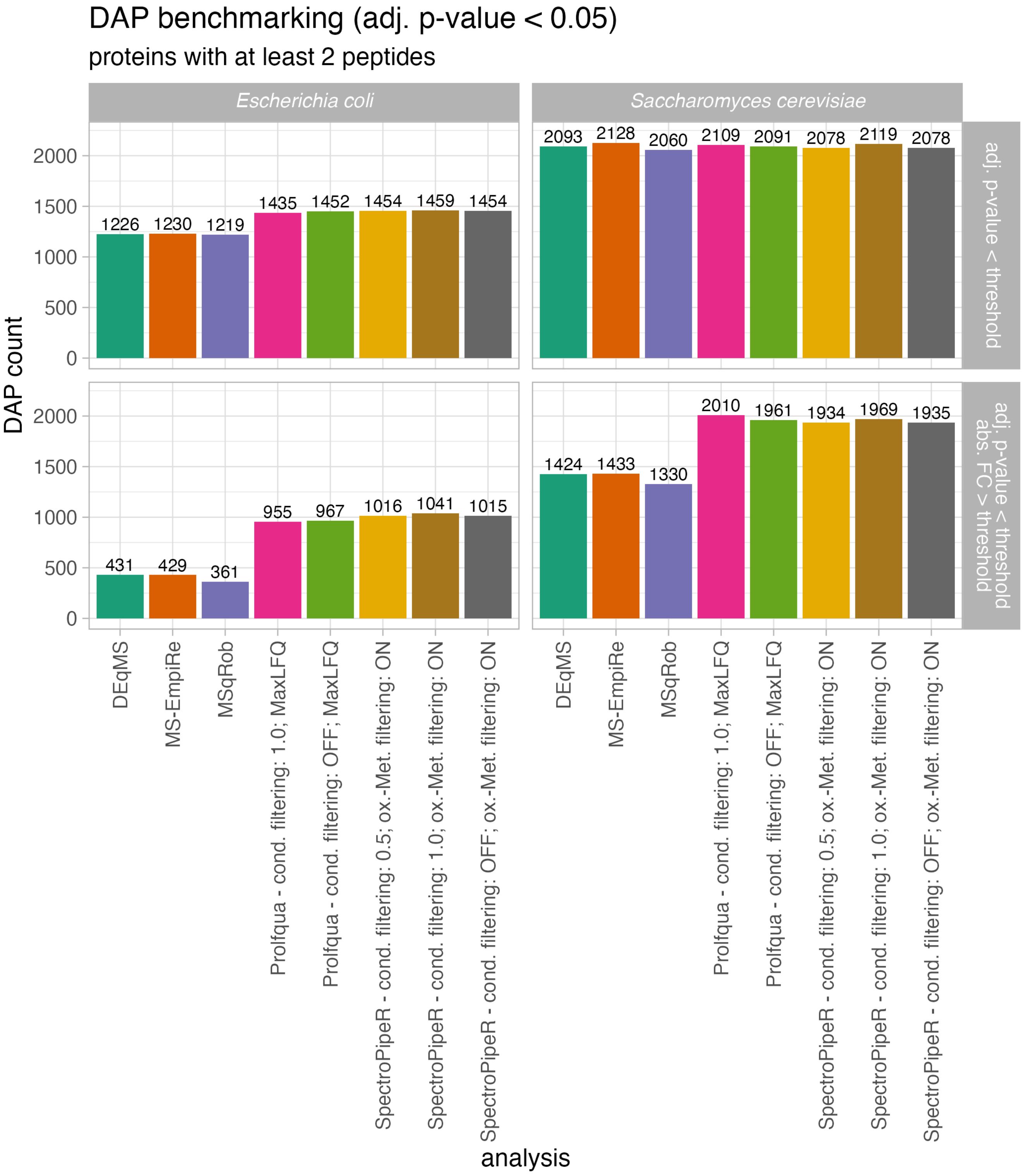

suppl. figure 13. *Benchmarking of various tools/settings was conducted using HYE species mix samples. The barplot illustrates the differentially abundant proteins (DAP) based on the statistical results of the different tools and settings with a adjusted p-value < 0.05. The plots are categorized by species, with the upper panel side showing only adjusted p-value and the lower panel side displaying both adjusted p-value and fold-change criteria (fold-change:* E.coli *= -3;* S. cerevisiae *= 1.5) for DAP counting. The x-axis depicts the analysis, and the y-axis shows the DAP count.*

The Differential Abundance Protein (DAP) analysis comparing various tools yielded comparable results for *E. coli* (expected absolute fold change of 4) but differed for MSqRob, MS-EmpiRe, and DEqMS. The difference was less pronounced when applying only an adjusted p-value filter to estimate the DAP count, which was also evident in the *S. cerevisiae* results. When using only an adjusted p-value filter to count the differentially abundant proteins, MSqRob, MS-EmpiRe, and DEqMS showed results comparable to Prolfaqua and SpectroPipeR. However, when applying fold-change filtering in addition to the adjusted p-value filtering, the rate dropped

drastically. This suggests that the fold-change calculation for these tools might not be optimal for the data used in this analysis. In summary, the Differential Abundance Protein (DAP) analysis demonstrated that SpectroPipeR yielded results that were comparable to, or even surpassed, those of other proteomics analysis tools.

6 SpectroPipeR processing time

Given that both the number of samples and the complexity of the sample significantly impact processing time, it is crucial to consider the computational demands of the algorithms employed. Specifically, more computationally intensive algorithms, such as the MaxLFQ algorithm (implemented in the iq package Pham *et al.* (2020)) for protein intensity calculation and the ROTS test for statistical analysis (implemented in the PECA package Suomi and Elo (2017)), require substantially longer processing times.

6.1 Processing time example

To estimate the processing time across different parameters, neat plasma sample measurements were utilized. From the sample set a defined number of samples were randomly drawn and used for the processing in SpectroPipeR with only one pairwise comparison.

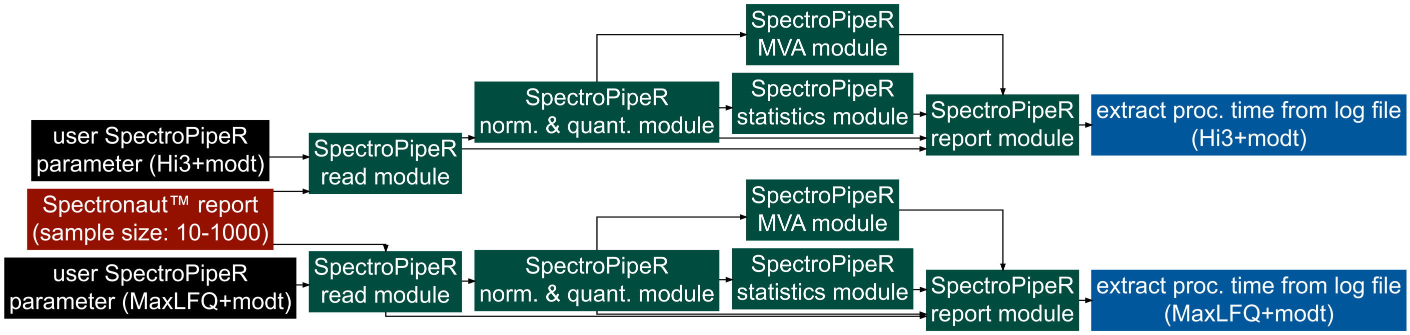

suppl. figure 14. *SpectroPipeR processing time example scheme*

The processing time was extracted from the log file after the completion of the read, normalization and quantification, multivariate analysis (MVA), statistics, and report modules (suppl. figure 14).

```
library(SpectroPipeR)
SpectroPipeR_analysis <- SpectroPipeR(file = "[input_file.tsv]",
                                      parameter = list(output_folder = "[output_folder]",
                                                       stat_test = "modt",
                                                       filter_oxidized_peptides = TRUE,
                                                       # Hi3 or MaxLFQ used for the processing example
                                                       protein_intensity_estimation = "MaxLFQ"
                                      ),
                                      condition_comparisons = cbind(c("[condition1]",
                                                                      "[condition2]")
                                                                    )
                                      )
```

computer setup used for the tests:

- processor: 3.6 GHz 8-Core Intel Core i9
- RAM: 64 GB
- operating system: MacOS 15.1.1
- R version: 4.4.1
- SpectroPipeR version: 0.4

number of samples & input file size:

Table 2: input file size table

sample size	file size [GB]
10	0.19
50	0.94
100	1.89
200	3.78
300	5.66
400	7.55
500	9.43
600	11.32
700	13.20
800	15.09
900	16.98
1000	18.86

sample set ID characteristics:

- number of ions without filtering = 6525
- number of peptides without filtering = 4260
- number of Protein groups without filtering = 469

6.1.1 Results of the processing time example

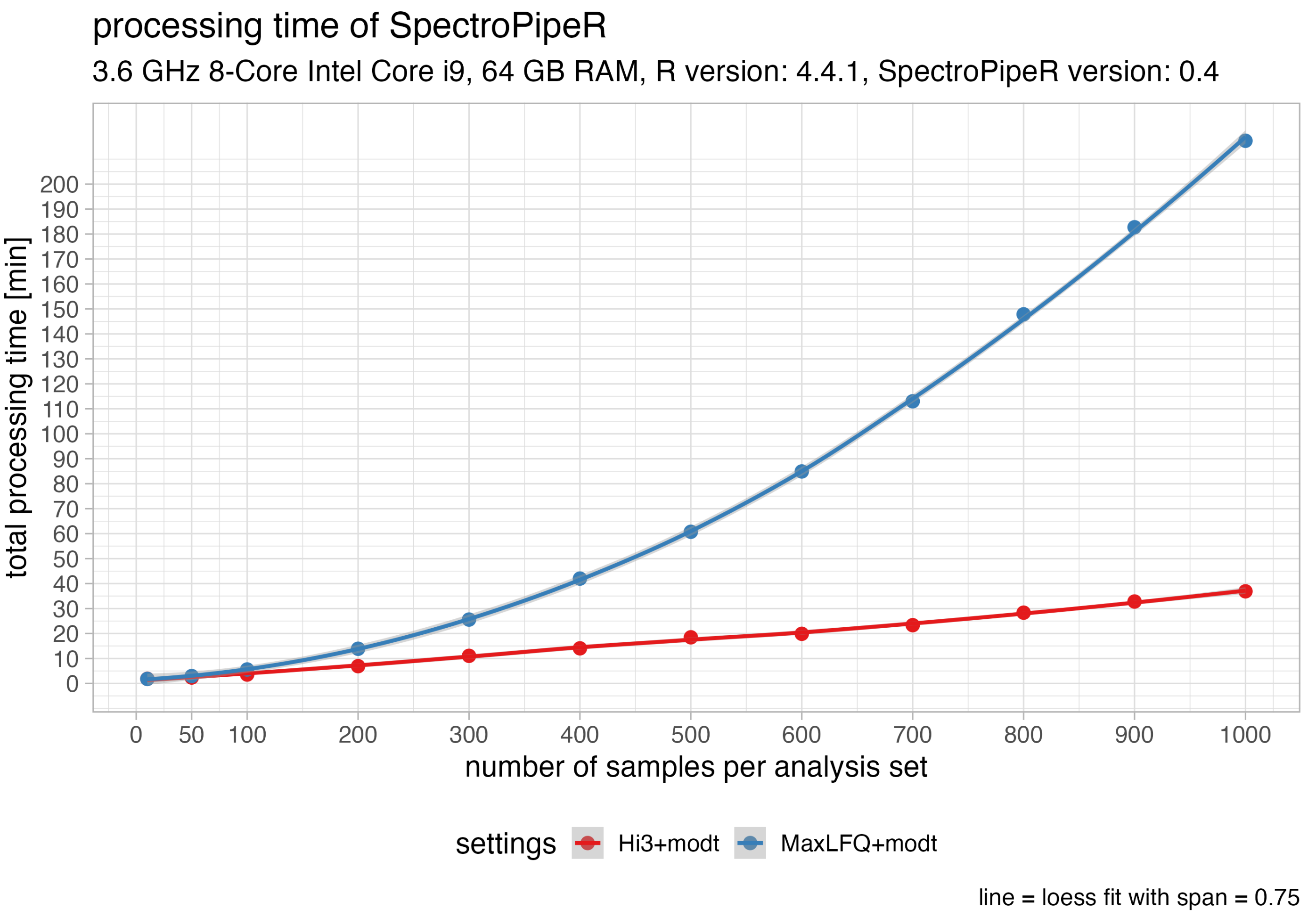

suppl. figure 15. *The plot illustrates examples of SpectroPipeR processing time. The x-axis represents the number of files in the input report, while the y-axis shows the final processing time, as extracted from the log file after the completion of processing.*

The results of the processing time test (suppl. figure 15) confirm that algorithms with lower computational complexity, such as the Hi3 protein intensity estimation method, are significantly less time-consuming during processing compared to more computationally intensive algorithms like MaxLFQ. Specifically, the Hi3 method demonstrated a marked reduction in processing time, making it a more efficient choice for large-scale proteomic analyses where time efficiency is critical. In contrast, the MaxLFQ algorithm, while offering high accuracy and robustness in quantification, requires substantially more processing time due to its complex computational demands. These findings highlight the importance of selecting the appropriate algorithm based on the specific requirements of the study.

7 Code snippets for SpectroPipeR

7.1 installation of SpectroPipeR

1. install R using https://cran.r-project.org.
2. optional: install RStudio IDE using https://posit.co/download/rstudio-desktop/
3. install Quarto CLI https://quarto.org/docs/get-started/
4. download and install SpectroPipeR

```
#install devtools
install.packages("devtools")
# install SpectroPipeR from github
devtools::install_github("stemicha/SpectroPipeR")
```

5. load the SpectroPipeR package and utilize the Spectronaut_export_scheme() function to create the necessary Spectronaut report scheme (SpectroPipeR_report.rs) in the output folder provided.

```
# load library
library(SpectroPipeR)
# output_location: path to output folder for the SpectroPipeR_report.rs Spectronaut report scheme
Spectronaut_export_scheme(output_location = "../SpectroPipeR_test_folder")
```

6. implement the SpectroPipeR_report.rs in Spectronaut® and export the Spectronaut® analysis report using the scheme

7.2 simple SpectroPipeR analysis

```
# load library
library(SpectroPipeR)

# use default parameters list
params <- list(output_folder = "../SpectroPipeR_test_folder")

# example input file // or path to your Spectronaut report (*.tsv)
example_file_path <- system.file("extdata", "SN_test_HYE_mix_file.tsv", package="SpectroPipeR")

# launch analysis
SpectroPipeR_analysis <- SpectroPipeR(file = example_file_path,
                                      parameter = params,
                                      condition_comparisons = cbind(c("HYE mix A","HYE mix B"))
                                      )
```

7.3 SpectroPipeR XIC plots

```
library(SpectroPipeR)
 # Spectronaut report path
 Spectronaut_report_path <- system.file("extdata/HYE_demo_data","HYE_demo_data_Report_SpectroPipeR.tsv",
                                                         package="SpectroPipeR")
# Spectronaut xicDB folder path
 Spectronaut_xicDB_path <- system.file("extdata/HYE_demo_data/XIC_DBs",package="SpectroPipeR")
# protein groups of interest
protein_groups <- c("P29311","P38720")
```

```r
# output folder path
output_path <- "../SpectroPipeR_test_folder/single_XIC_plots"

 # extracting and plotting of XIC
 XIC_plot_module(Spectronaut_report_path = Spectronaut_report_path,
                                Spectronaut_xicDB_path = Spectronaut_xicDB_path,
                                protein_groups = protein_groups,
                                output_path = output_path,
                                export_csv_files = TRUE,
                                number_of_cores = 2
 )
```

7.4 SpectroPipeR gui

```r
# load library
library(SpectroPipeR)

# load SpectroPipeR GUI
SpectroPipeR_ui()
```

References

Chen,M. and Cook,K.D. (2007) Oxidation Artifacts in the Electrospray Mass Spectrometry of Aβ Peptide. *Analytical Chemistry*, **79**, 2031–2036.

Hains,P.G. and Robinson,P.J. (2017) The Impact of Commonly Used Alkylating Agents on Artifactual Peptide Modification. *Journal of Proteome Research*, **16**, 3443–3447.

Koopmans,F. *et al.* (2022) MS-DAP Platform for Downstream Data Analysis of Label-Free Proteomics Uncovers Optimal Workflows in Benchmark Data Sets and Increased Sensitivity in Analysis of Alzheimer's Biomarker Data. *Journal of Proteome Research*.

Pham,T.V. *et al.* (2020) iq: an R package to estimate relative protein abundances from ion quantification in DIA-MS-based proteomics. *Bioinformatics*, **36**, 2611–2613.

Reder,A. *et al.* (2023) MassSpecPreppy—An end-to-end solution for automated protein concentration determination and flexible sample digestion for proteomics applications. *PROTEOMICS*.

Suomi,T. and Elo,L.L. (2017) Enhanced differential expression statistics for data-independent acquisition proteomics. *Scientific reports*, **7**, 5869.

Zang,L. *et al.* (2012) Residual metals cause variability in methionine oxidation measurements in protein pharmaceuticals using LC-UV/MS peptide mapping. *Journal of Chromatography B*, **895**, 71–76.
